# Inhibition of Both HIV-1 Reverse Transcription and Gene Expression by a Cyclic Peptide that Binds the Tat-Transactivating Response Element (TAR) RNA

**DOI:** 10.1371/journal.ppat.1002038

**Published:** 2011-05-19

**Authors:** Matthew S. Lalonde, Michael A. Lobritz, Annette Ratcliff, Mastooreh Chamanian, Zafiria Athanassiou, Mudit Tyagi, Julian Wong, John A. Robinson, Jonathan Karn, Gabriele Varani, Eric J. Arts

**Affiliations:** 1 Department of Biochemistry, Case Western Reserve University, Cleveland, Ohio, United States of America; 2 Department of Molecular Biology and Microbiology, Case Western Reserve University, Cleveland, Ohio, United States of America; 3 Department of Chemistry and Department of Biochemistry, University of Washington, Seattle, Washington, United States of America; 4 Department of Chemistry, University of Zurich, Zurich, Switzerland; 5 Division of Infectious Diseases, Department of Medicine, Case Western Reserve University, Cleveland, Ohio, United States of America; Northwestern University, United States of America

## Abstract

The RNA response element TAR plays a critical role in HIV replication by providing a binding site for the recruitment of the viral transactivator protein Tat. Using a structure-guided approach, we have developed a series of conformationally-constrained cyclic peptides that act as structural mimics of the Tat RNA binding region and block Tat-TAR interactions at nanomolar concentrations *in vitro*. Here we show that these compounds block Tat-dependent transcription in cell-free systems and in cell-based reporter assays. The compounds are also cell permeable, have low toxicity, and inhibit replication of diverse HIV-1 strains, including both CXCR4-tropic and CCR5-tropic primary HIV-1 isolates of the divergent subtypes A, B, C, D and CRF01_AE. In human peripheral blood mononuclear cells, the cyclic peptidomimetic L50 exhibited an IC_50_ ∼250 nM. Surprisingly, inhibition of LTR-driven HIV-1 transcription could not account for the full antiviral activity. Timed drug-addition experiments revealed that L-50 has a bi-phasic inhibition curve with the first phase occurring after HIV-1 entry into the host cell and during the initiation of HIV-1 reverse transcription. The second phase coincides with inhibition of HIV-1 transcription. Reconstituted reverse transcription assays confirm that HIV-1 (−) strand strong stop DNA synthesis is blocked by L50-TAR RNA interactions *in-vitro*. These findings are consistent with genetic evidence that TAR plays critical roles both during reverse transcription and during HIV gene expression. Our results suggest that antiviral drugs targeting TAR RNA might be highly effective due to a dual inhibitory mechanism.

## Author Summary

The HIV-1 transactivator protein (Tat), together with the elongation factor P-TEFb binds to an HIV-1 RNA secondary structure in the 5′-UTRs of nascent viral mRNAs (TAR) and promotes transcription elongation. This process has been an attractive target for drug development but previous inhibitors that bind either Tat or TAR have been plagued by poor inhibition of virus replication, limited cell penetration, and off-target effects. In this article, we describe a series of rationally designed cyclic peptides that block Tat-TAR interactions. L50, the most potent of these compounds, inhibits a wide range of HIV-1 strains from around the world. Remarkably, L50 inhibits two distinct steps in the HIV-1 lifecycle. As expected, L50 inhibits Tat-dependent HIV-1 transcription, but the majority of its anti-HIV activity is due to a block in reverse transcription, i.e. synthesis of the proviral DNA from the RNA genome. L50 inhibition of reverse transcription reveals an important role for TAR RNA during reverse transcription as well as providing one of first examples of a drug with a dual mechanism of action.

## Introduction

Highly active antiretroviral therapy (HAART) has led to a dramatic increase in the longevity of patients infected with HIV [1]. Unfortunately, even the most effective therapy does not completely eradicate the virus and active viral replication resumes immediately after treatment interruption [2]. The emergence of drug resistance further complicates antiviral therapy and can lead to treatment failure [3], underscoring the continuing need to develop new HIV antivirals with novel targets and mechanisms of action [4].

Tat, a viral encoded transcriptional activator, and its cellular co-factor, the transcription elongation factor-b (P-TEFb) are recruited to the elongating RNA polymerase II (RNAP II) through interactions with the trans-activation responsive element (TAR), a 59­nucleotide RNA found at the 5′ end of all viral transcripts (for reviews, see [5], [6]). Assembly of this complex activates P-TEFb subunit CDK9 kinase activity [7]. CDK9-mediated phosphorylation of RNAP II and Spt5 (a subunit of the DRB-sensitivity inducing factor (DSIF)) directly enhance transcriptional elongation [8]–[10] as well as dissociation of repressive NELF factor(s) [11], allowing more efficient RNAP II promoter clearance. The Tat:P-TEFb crystal structure, reported by by Tahirov et al. [12], reveals how these proteins associate and the concomitant conformational changes this interaction induces in the CycT1:CDK-9 complex. While P-TEFb is utilized widely for transcription of many genes, the interaction between Tat and TAR is unique to lentiviruses. Drugs targeting Tat and/or TAR are expected to both block HIV-1 replication during the acute phase of HIV-1 infection and to prevent virus emergence from latency [13]. A variety of candidate small molecule inhibitors of either HIV transcription, or more specifically, the Tat-TAR interaction, have been identified during the last 15 years [9], [10], [14]–[19]. Unfortunately none of these compounds were sufficiently potent and/or selective to progress beyond phase I clinical trials. Linear polypeptide analogues have been shown to block the Tat-TAR interaction by binding to TAR RNA [16], [17], [20], [21], but their conformational flexibility also allowed promiscuous binding to host RNAs and non-specific blocks to viral infectivity [22], [23]. Using constrained cyclic peptidomimetics designed to mimic the antiparallel ß-sheet in the Tat RNA binding domain we identified a series of competitive inhibitors of the Tat-TAR interaction with improved affinity and selectivity compared to the linear peptides [24]–[27].

In this study, we investigated the antiviral mechanism(s) of these compounds. Peptidomimetics in this compound series were cell permeable, had low cytotoxicity, and inhibited viral replication of a diverse HIV-1 isolate panel in both primary human cells and immortalized cell lines with low micromolar half-maximal inhibitory concentration (IC_50_) values. In contrast to other flexible linear peptide inhibitors of TAR binding, constrained peptidomimetics had no effect on HIV-1 entry [22], [23]. Surprisingly, inhibition of viral transcription did not account for the full antiviral activity of these compounds. Timed drug addition experiments then demonstrated that the peptidomimetics have a dual mechanism of action, blocking both HIV-1 reverse transcription (an early infection event) and HIV-1 proviral RNA transcription (a later event).

## Results

### Inhibition of HIV-1 replication by Tat peptidomimetics

**Figure 1:**
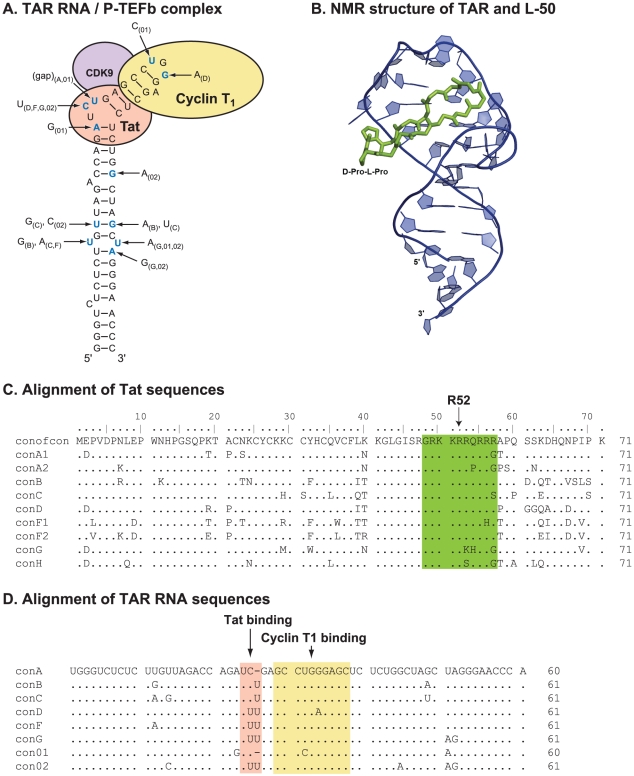
The structure and sequence of HIV-1 Tat/TAR RNA. A schematic of the secondary RNA structure of HIV-1 TAR is presented in panel (**A**) to describe the relative positioning/interaction with HIV-1 Tat, Cyclin T1, and CDK9. The sites of nucleotide sequence variability among HIV-1 isolates are indicated by the arrows. The bracketed letter or number associated with the polymorphism defines the HIV-1 subtype or circulating recombinant form, CRF (respectively). (**B**) The NMR structure of HIV-1 TAR complexed with a small molecule (RBT203) is shown in a space filling model (PDB file 1UUD in ball-and-stick). (**C**) Amino acid alignment of the consensus sequences of HIV-1 Tat proteins from all group M HIV-1 subtypes, which are responsible for the worldwide epidemic. (**D**) The consensus nucleotide sequence of the HIV-1 TAR RNA element in the various HIV-1 subtypes.

Linear peptide derivatives have been used previously to target TAR RNA and block Tat binding [16], [17], [20], [21] but their utility was limited by conformational flexibility which allowed non-specific RNA binding. To avoid this problem we have developed conformationally constrained mimics of HIV-1 Tat [24]–[27] based on the structure of the BIV (Bovine Immunodeficiency Virus) Tat-TAR complex [28], [29] (Figure 1). Specifically, we constructed a series of ß-hairpin mimics with 12-residue loops on a heterochiral D-Pro-L-Pro dipeptide scaffold. This D-Pro-L-Pro scaffold strongly favors a type-II' ß-turn backbone conformation, similar to that found in the Tar RNA binding domain [24]–[27]. Assayed in-vitro, several members of this series, L-50, L-51, and L-22, bound HIV-1 TAR with nanomolar affinity (K_d_ = 1, 5, and 30 nM, respectively) [25], [26].

**Figure 2:**
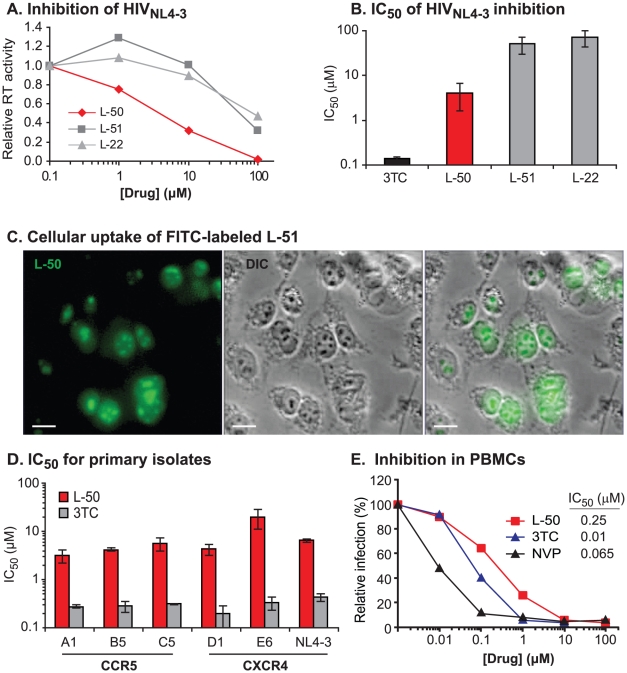
Testing the susceptibility of HIV-1 to inhibition by Tat peptidomimetics. (**A**) Comparison of the relative inhibition of the HIV-1 NL4-3 laboratory strain by three lead Tat peptidomimetics. Virus production in cell free supernatant was measured using an endogenous RT assay. (**B**) The concentration for 50% inhibition (IC_50_) of HIV-1 NL4-3 was calculated for the lead Tat peptidomimetics and for the reference antiretroviral, 3TC. (**C**) Cell penetration (hT4R5 fibroblasts) and nuclear localization of fluorescein-labeled L-51 peptide analyzed by confocal microscopy. (**D**) L-50 inhibition of viral replication of three primary CCR5-tropic HIV-1 isolates measured in U87.CD4.CCR5 cells (A1 is the subtype A Rawandan isolate A1-92RW009, B5 is the subtype B isolate B5-91US056 from the USA and C5 is the subtype C isolate C5-97ZA003 from South Africa) and of CXCR4-tropic strains (D1 is the Ugandan subtype D isolate D1-92UG021, E6 is the subtype A/E circulating recombinant isolate CRF01_AE from Thailand) as well as the laboratory strain HIV-1_NL4-3_ measured in U87.CD4.CXCR4 cell cultures. The IC_50_ values for L50 and 3TC were calculated from drug susceptibility curves (Figure S1). (**E**) Inhibition of HIV NL4-3 was also measure in human peripheral blood mononuclear cells stimulated with PHA/IL-2. The nucleoside RT inhibitor, 3TC or the non-nucleoside RT inhibitor, nevirapine (NVP) were used as controls. Virus production was measured at 8 and 10 days post-infection by the RT activity in the supernatant (cpm/mL).

As shown in Figure 2A, the three Tat peptidomimetics L-50, L-51 and L-22, were able to inhibit replication of laboratory-adapted HIV-1 strain NL4-3 (HIV-1_NL4-3_) in U87 cells expressing the CD4 receptor and CXCR4 coreceptor (U87.CD4.CXCR4 cells). The relative potency of the compounds correlated with their TAR binding activity [26]. L-50, which had the highest affinity for TAR, inhibited HIV-1_NL4-3_replication with an IC_50_ value of 4.1 µM, which was about 10-fold more potent than either L-51 or L-22 (Figure 2B). The activity of L-50 was approximately 10-fold lower than the licensed antiretroviral drugs 3TC (IC_50_ = 0.12±0.03 µM on U87 cells; Figure 2B) as well as Enfuvirtide (IC_50_ = 0.1 to 0.5 µM in U87 cells) [30], a polypeptide retroviral entry inhibitor which does not require cellular uptake [31].

### Cellular uptake

The sequences of the peptidomimetics are derived from the HIV-1 Tat basic domain (amino acids 48–57) which, in addition to RNA binding, permits the protein to transverse cellular membranes [32]–[35]. Since known cell-penetrating peptides have flexible structures, we tested whether the constrained peptides retained cell-penetrating properties. A fluorescein-labeled conjugate of polypeptide L-51, prepared by coupling a commercially available fluorescein-diacetate tag to a hydrazine-derivative of L-51, was rapidly internalized by living 293T fibroblasts (Figure 2C). The peptidomimetic accumulated in the nucleus with a substantial fraction in the nucleoli, resulting from their semblance to nuclear and nucleolar localization signals [36], [37].

### Cytotoxicity

L-50 toxicity was compared to that of enfuvirtide (T20), another anti-HIV-1 inhibitor polypeptide. U87.CD4.CXCR4 cells were exposed to increasing concentrations of the peptides (maximal of 500 µM) for ten days and cell viability was measured using Alamar blue staining. Cell viability was similar to control conditions under all conditions, including the highest L-50 or enfuvirtide concentration tested (500 µM; data not shown), consistent with previous studies experiments with T20 [31].

### Inhibition of multiple HIV-1 clades by the Tat peptidomimetics

Although pre-clinical development of antiviral drugs typically involves initial optimization with subtype B laboratory HIV-1 strains, HIV-1 subtypes A, C, D, and CRF01_AE account for over 85% of the global epidemic whereas subtype B comprises less than 10% of present infections [38]. Thus, the long-term utility of any antiretroviral lies in its ability to inhibit diverse primary HIV-1 isolates of all subtypes - particularly those strains that utilize the dominant co-receptor (CCR5) for entry. As shown in Figure 2D, L-50 effectively inhibited a panel of CCR5-tropic and CXCR4-tropic primary HIV-1 isolates including representatives of subtypes A, B, C, D, and CRF01_AE (Figure S1A, B). Aside from strain E6-CRF01_AE, the mean IC_50_ value for L-50 was 4.73±1.33 µM (Figure 2D). Cross-clade HIV-1 inhibition profiles are important attributes for potential Tat-TAR inhibitors, since there is considerable genetic diversity in both the R52 domain of the Tat protein (Figure 1C) as well as its TAR RNA target sequence in the HIV-1 LTR (Figure 1D). Furthermore, previous studies using a linear peptoid Tat-TAR interaction inhibitor in vitro showed that the principal antiviral activity was due to blocking the CXCR4 receptor, preventing virus entry rather than viral transcription [27]. The broad-spectrum of antiviral activity against multiple clades and viruses that utilize either CCR5 or CXCR4 co-receptors suggests that the constrained peptidomimetics do not act at viral entry.

Early-stage drug candidates often display efficient HIV-1 inhibition in cell lines but lack potency in primary CD4+ T lymphoctyes. In the case of L-50, the efficiency of HIV-1_NL4-3_ inhibition was actually increased in human PBMCs (IC_50_ 0.25 µM) compared to U87.CD4.CXCR4 cells (IC_50_ 4.1 µM) (Figure 2E).

### Inhibition of Tat-dependent transactivation in cell-free transcription systems

**Figure 3:**
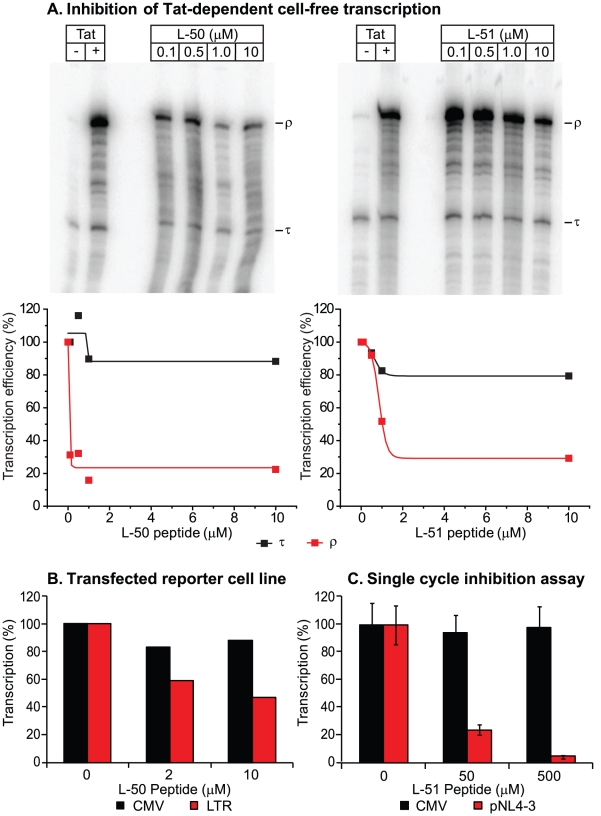
L50 inhibition of Tat-dependent transactivation in cell-free, reconstituted transcription system and in transfected cells. DNA templates carrying the HIV promoter were transcribed in the presence or absence of recombinant Tat protein (+Tat) and increasing concentrations of the Tat peptidomimenic (L-50, left panel; L-51, right panel) (**A**); ρ identifies full-length transcripts and τ identifies transcripts ending at the terminator sequence inserted proximal to the promoter. Relative transcription levels of the Tat-dependent full-length transcript ρ decreased by 4-fold with the Tat peptidomimetic while the Tat-independent τ product remained almost unaffected. (**B**) 293T cells were transfected with the plasmids pLTR-luc (LTR) + Tat or pcDNA.LUC (CMV), where the luciferase gene was under the control of the HIV-1 LTR or CMV promoters, respectively. Cells were treated with either 2 or 10 µM L50. Relative light units, based on luciferase expression, were reported as percentages of the no drug controls. (**C**) L-50 was added to 293T cells transfected infectious molecular clone (pNL4-3) while expression of 293T cells transfected with luciferase reporter provided the control. Virus production from the 293T cells transfected with pNL4-3 was monitored by RT activity and expressed as a percent transcription.

We next tested whether the peptidomimetics could inhibit Tat function in a cell-free transcription assay (Figure 3). Cell free transcription assays have been extensively used in previous studies of compounds capable of inhibiting Tat [8], [15], [39], [40]. Briefly, nuclear extracts are incubated with a DNA template harboring the HIV-1 long terminal repeat (LTR; contains the proviral promoter and the TAR region) either in the presence or absence of recombinant Tat protein (Figure 3A). A terminator sequence inserted downstream of the promoter provides a selective and effective block to RNAP II elongation in the absence of Tat. As shown in Figure 3A, transcripts that are unable to traverse the terminator (labeled τ) accumulated in the absence of Tat, whereas full-length transcript (labeled ρ) production increased over 20-fold when Tat was added. Since the levels of short transcripts (τ) should remain unaffected when Tat is inhibited, these transcripts provide an effective internal control for non-specific inhibition of transcription. As shown in Figures 3A, addition of increasing concentrations of the inhibitory peptides L-50 and L-51 decreased synthesis of full-length transcripts (ρ) with an IC_50_ of 50 nM and 500 nM, Under these conditions the production of short transcripts was largely unaffected. Thus, the Tat peptidomimetics were potent inhibitors of Tat-mediated transcriptional elongation in cellular extracts.

### Inhibition of transactivation *in vivo*

**Figure 4:**
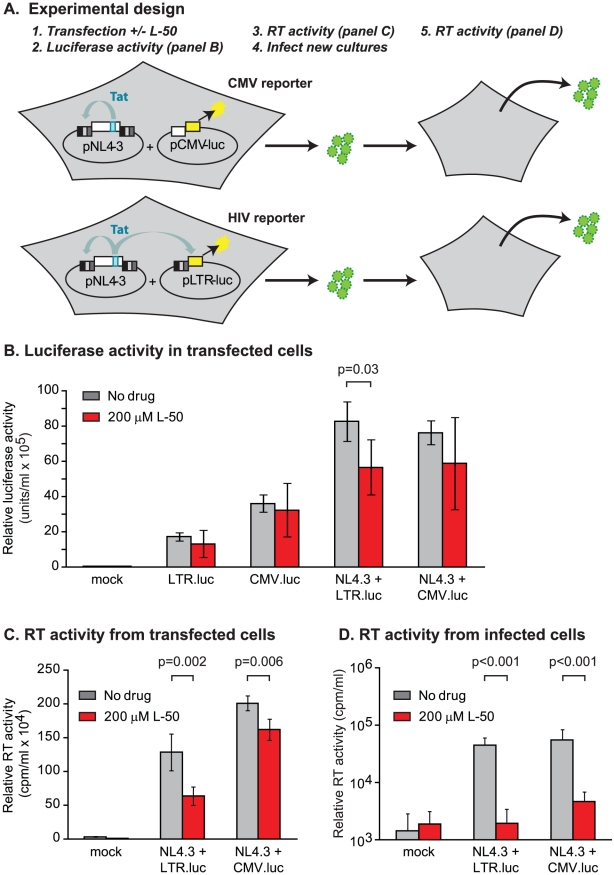
L50 effects on Tat-mediated transactivation of mRNA transcription in cell lines. (**A**) A schematic of the dual transfection of pNL4-3 and pcDNA.LUC (CMV) or pLTR-luc in 293T cells. Luciferase (in lysates) (**B**) and RT activity (in cell-free supernatants) (**C**) was measured 72 h following transfection/drug treatment of 293T cells. Supernatant from 293T transfections conditions were used to infect U87.CD4.CXCR4 cells. (**D**) Virus production at 10 days post infection was measured by RT activity.

In order to evaluate the impact of the Tat peptidomimetics on HIV-1 transcription in vivo, we used three distinct reporter assay systems (Figures 3 & 4). First, we measured inhibition of transcription of stably transfected luciferase reporter genes under the control of the HIV-1 LTR and a control CMV promoter (Figure 3B). The addition of Tat increased the levels of the luciferase reporter gene expression by more than 30-fold in the case of LTR promoter while the transcription levels of the CMV promoter remained unaffected (data not shown). Treatment with 10 µM L-50 reduced Tat-mediated transactivation by 53%, but only reduced transcription from the control CMV promoter by 12% (Figure 3B).

Inhibition of HIV transcription was also measured in a single-cycle growth assay (Figure 3C). 293T cells were cotransfected with an infectious molecular clone HIV_NL4-3_ and a control vector carrying a luciferase reporter gene downstream of the CMV promoter. 293T cells support Tat-mediated HIV-1 transcription and virus production but are resistant to new rounds of virus production since they lack entry receptors. Addition of 50 µM L-50 peptide inhibited production of the viral RNA by 76% but as expected for a specific Tat-dependent transcription inhibitor, CMV promoter-driven luciferase activity reduced by less than 5% by L-50. Thus, inhibition of HIV-1 transcription by L-50 in both assays arises from blocks to transcription imposed by a disruption of the Tat-TAR interaction.

In the third assay, 293T cells were co-transfected with a reporter plasmid carrying a luciferase reporter downstream of the HIV-1 LTR promoter (pLTR.LUC) alone or in combination with the Tat-encoding infectious molecular clone HIV-1_NL4-3_. In control experiments, cells were co-transfected with a reporter plasmid carrying the luciferase gene downstream of the CMV promoter (pCMV.LUC) alone or in combination with HIV-1_NL4-3_ (Figure 4A). L-50 did not inhibit luciferase expression in cells transfected with either reporter plasmid alone (Figure 4B). Thus, although pLTR.luc produces the TAR RNA element as part of the luciferase mRNA, transcription is not blocked by L-50 in the absence of Tat. When cells were co-transfected with HIV-1_NL4-3_, Tat expression resulted in a ∼6.3-fold increase in luciferase expression. L-50 (200 µM) resulted in a comparatively modest (1.7-fold), but significant (p = 0.03, two-tailed T test, N = 5), decrease in luciferase expression (Figure 4B). As expected, CMV promoter-driven luciferase expression was not significantly inhibited by L-50.

In addition to measuring luciferase activity, we also measured viral production to provide an additional measurement of the antiviral activity of the compounds. It is important to note that, although luciferase expression is not LTR-driven in cells transfected by the pCMV.luc control plasmid, HIV-1_NL4-3_-encoded virion production is LTR-dependent in both sets of transfected cells. Treatment with 200 µM L-50 mediated a modest, but reproducible reduction in HIV-1 virion expression (Figure 4C). These results were consistent with measurements wherein L-50 inhibited transcription from LTR-driven cassettes (Figure 4B). The slightly greater inhibition of virus production from the pLTR.luc transfected cells by L-50 compared to the pCMV.luc transfected cells is likely due to competition between the viral and reporter expression cassettes for limited Tat protein (Figure 4C).

Thus we were able to demonstrate Tat-mediated transcriptional inhibition by L-50 in three distinct assays. However, in all these assays we were only able to detected a 2-fold reduction of RNA or virus production, which is considerably less than the 10- to 100-fold HIV-1_NL4-3_ replication block in the multiple-cycle replication assays described above. Several possible explanations for this discrepancy might be: 1) virus released from the transfected cells were no longer replication-competent, 2) that L-50 has viricidial activity, or 3) that L-50 inhibited virus replication at additional steps in the viral life cycle prior to the onset of gene expression.

To test whether the virus released from transfected cells could be inhibited by L-50 under conditions of multiple-cycle growth, virus-containing supernatants from 293T transfections (containing L-50) were used to infect U87.CD4.CXCR4 cells (which support multiple rounds of HIV-1 replication) and viral progeny were measured 10 days post-infection. No measurable viral progeny were produced by viruses which were made in the presence L-50 (Figure 4D). Removal of L50 from virus was attempted by pelleting virus from supernatant of L50-treated cells transfected with NL4-3. It is important to note that virus production was already decreased by L50 at least 50% from transfected cells.

While the results of that experiment (Figure 4D) imply that L-50 inhibits viral egress through the target cells at some event prior to viral transcription, those data could not rule out virucidal activity. A cell-free preparation of HIV-1_NL4-3_ virions was incubated for 2 h with or without L-50 (50 µM or 500 µM). After pelleting virus to remove drug, we found similar viral infectivity with and without L-50 treatment suggesting that this drug was not a viricide (Figure S2). The strong implication of this experiment is that L-50 inhibited HIV replication by another mechanism during the replication cycle.

### Time-dependent drug addition kinetics

**Figure 5:**
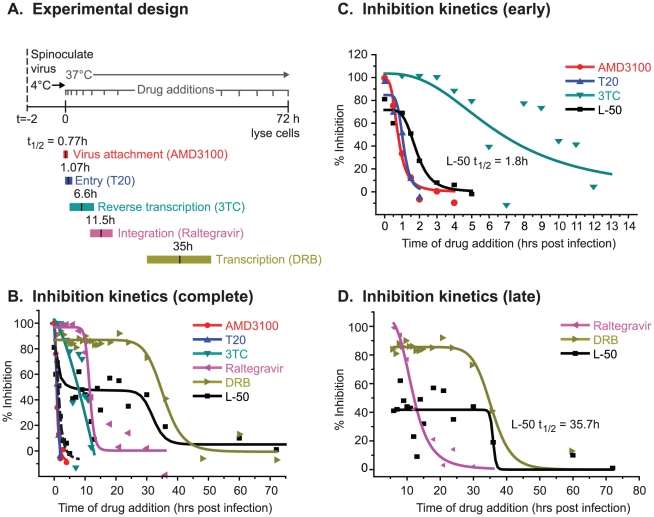
Time-of-drug-addition experiment during synchronized HIV-1 infections. Panel (**A**) provides a schematic representation of the experimental protocol, including the spinoculation step for synchronized virus infection and timing of drug additions over the 72 h time course. The relative time frame associated with each retroviral step is defined based on the time frame of sensitivity to the drug that blocks that particular replication step. Panel (**B**) plots the level of inhibition mediated by a specific drug added at a specific time post infection. Virus production, regardless of the timing of drug addition, was measured by luciferase activity at 72 h post infection. Curves are fitted to % inhibition mediated by the timed addition of each drug. Inhibition by a drug is absent after the completion of the specific step known to be a target of that drug. For example, AMD3100 bind CXCR4 and prevents HIV-1 entry; thus, inhibition of HIV-1 by AMD3100 is not observed if the drug is added >2 h post infection, i.e. after the HIV-1 entry step is complete. A biphasic curve was fitted to inhibition mediated by timed addition of L50. The first 12 hours of the timed drug addition was magnified to examine the inhibition of HIV-1 entry and reverse transcription (panel **C**; early events). These early steps of HIV-1 replication were removed from the plots in panel **D** (late events) to focus on the timed inhibition by integration and transcription inhibitors. The times of drug addition that maintains 50% inhibition (t_1/2_) are shown for each drug in the insets. All time-of-drug-inhibition experiments were performed in triplicate which resulted in a 10–15% variance in the inhibition levels. Error bars are not shown to prevent figure congestion.

The preceding experiments show that, although L-50 weakly inhibits HIV transcription *in vivo*, this activity does not account for the majority of its antiviral activity. To identify the key step(s) in the virus replication cycle that are inhibited by L-50, time-of-drug-inhibition experiments were performed (Figure 5). For each measurement, virus was added to cells at time 0 and HIV-1 gene expression (based on luciferase expression) was assayed at 72 h post infection. The HIV-1_NL4-3_virus employed in the experiment shown in Figure 5 carried the firefly luciferase gene inserted between the *env* and *nef* genes [41]. Luciferase expression was therefore dependent on LTR-mediated transcription and was used as a readout for viral replication. Comparable results were obtained in experiments when virus production was measured by RT activity (data not shown). Virus replication was limited to a single round by the addition of the HIV-1 protease inhibitor saquinavir, at 2 h post infection in each experiment.

The time of addition assay was calibrated using HIV inhibitors which selectively inhibit distinct steps in the viral replication cycle (Figure 5A). All drugs were added at intervals and, as the infection progressed, each drug became ineffective at a time consistent with the completion of their targeted infection event (Figure 5B). Additions of AMD3100, a CXCR4 antagonist which prevents HIV-1 co-receptor attachment, became ineffective very soon after infection (t_1/2_ = 0.77 h; Figure 5B & C). The viral fusion inhibitor Enfuvirtide (or T20) remained effective at slightly later additions (t_1/2_ = 1.0 h), consistent with a fusion event occurring after receptor binding. Inhibition by 3TC is slowly lost over 12 h (t_1/2_ = 6.6 h; Figure 5D), i.e. throughout the time required to complete reverse transcription (Figure 5B), consistent with previous results for this drug [42].

The integration inhibitor Raltegravir and the transcription inhibitor 5,6-dichloro-1-ß-D-ribobenzimidazole (DRB), blocked HIV-1 replication with significantly delayed kinetics compared to the entry and reverse transcription inhibitors (Raltegravir t_1/2_ = 11.5 h; DRB t_1/2_ = 31.1 h; Figure 5B & D). It is worth noting that data with DRB, which is a potent inhibitor of the CDK9 subunit of P-TEFb, can be considered to behave analogously to a potential Tat inhbititor, since P-TEFb is strictly required for Tat-dependent HIV transcription [43], [44].

When L-50 was added either prior to infection or at various time points following infection (every 30 minutes for the first 2 h, every hour for the next 13 hours, and then every 3–6 hours thereafter), we observed an unusual, biphasic inhibition profile over the 72 hour time course (Figure 5B). There was an initial inhibitory phase at approximately 2 h post infection (t_1/2_ = 1.8 h) when L-50 was able to block nearly 100% of viral replication. Thus, L-50 inhibits a step immediately following virus entry within the time window of the first stages of HIV-1 reverse transcription. When L-50 was added during the next 20 hours (t_1/2_ of 35.7 h), i.e. during the period after reverse transcription and subsequent to completion of proviral integration, there was a second phase of viral inhibition. The timing of this second phase was similar to the inhibitory kinetics of transcription inhibitor DRB (35.3 h; Figure 5E). Thus, L-50 blocks both an early post-entry step during HIV-1 replication as well as Tat-mediated transactivation of HIV-1 transcription.

### L-50 does not block HIV-1 entry

**Figure 6:**
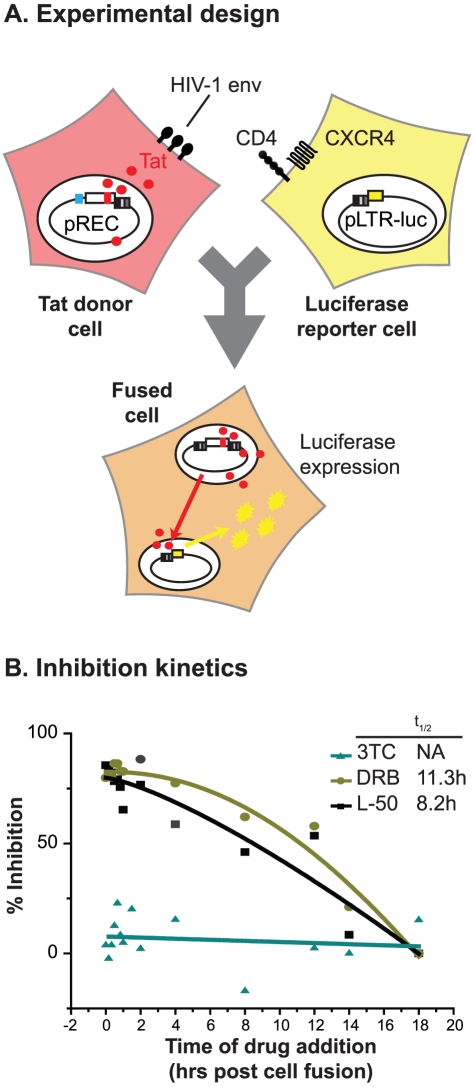
Time-of-drug-addition experiments during synchronized cell-to-cell fusion. (**A**) A cell-to-cell fusion was mediated by an effector 293T cell, expressing HIV-1 Env gp120/gp41 (via pREC nfl transfection), binding to CD4 and CXCR4 receptors on a target U87 cell. The luciferase reporter is only expressed after HIV-1 Tat and Rev migrate from the effector cytoplasm to the target nucleus in the fused cell and transactivates/rescues LTR-driven mRNA expression. (**B**) The timing of 3TC, DRB, and L50 inhibition was measure in this synchronized cell-to-cell fusion where a smaller number of transfected 293T cells are pelleted onto adherent U87.CD4.CXCR4 cells. Luciferase expression is measure 48 h post cell fusion. The times of drug addition that maintains 50% inhibition (t_1/2_) are shown for each drug in the insets. Triplicate measurements resulted in a 10–15% variance in the inhibition levels.

The kinetic experiments shown in Figure 5, demonstrated that L-50 primarily inhibits HIV-1 replication at a step immediately following entry (t_1/2_ = 1.8 h) but prior to the inhibition of reverse transcription by 3TC (t_1/2_ = 6.6 h). The timing of these events is consistent with L-50 inhibiting either a very early step in reverse transcription or an entry step following formation of the gp41 pre-hairpin intermediate, i.e. the Enfuvirtide-sensitive state, prior to cell-viral membrane mixing and pore formation. To distinguish between these two possibilities, we performed a second time-of-drug inhibition experiment using cell-to-cell fusion to introduce Tat and activate an already integrated provirus (Figure 6A). In this assay, effector 293T cells were transfected with pREC.NFL, a CMV-driven expression plasmid carrying an HIV-1 provirus and lacks the HIV-1 5′-LTR sequence [45]. Transcription of the proviral construct produces all the HIV-1 proteins, including the gp120-gp41 envelope glycoprotein, which is expressed on the cell membrane and is required to induce cell fusion with the U87.CD4.CXCR4 target cells. Since the pREC.NFL vector lacks a5′-LTR, none of the transcripts produced in the effector cells can serve as templates for reverse transcription [45]. The U87.CD4.CXCR4 target cells were transfected with pDM128-LTR-fluc2 (see Materials and Methods) to provide a Tat- and Rev-dependent reporter. After fusion of the two cells, the HIV-1 Tat (and Rev) proteins migrate into the nucleus of the target cell, and stimulate luciferase production (Figure 6A). Thus, this assay is sensitive to inhibitors of membrane fusion and viral entry and inhibitors of viral transcription, but insensitive to inhibitors of reverse transcription.

Cell-to-cell fusion was initiated by pelleting the transfected 293T cells on to monolayers of U87.CD4.CXCR4 cells at 4°C. The drugs (L-50, 3TC, or DRB) to be tested were added at various time points, similar to the timed-drug-addition experiment described above (Figure 6A). Tat/Rev-dependent luciferase expression was measured in the cell lysates 72 h post fusion. This cell-to-cell fusion assay is highly sensitive to inhibition by both T20 and AMD3100 (data not shown) indicating that cell-to-cell fusion is both receptor-dependent and mechanistically identical to HIV-1 entry into a cell [46]. Because cell-to-cell fusion is not followed by reverse transcription or integration, 3TC was unable to inhibit reporter gene activation at any time (Figure 6B). In contrast to the time-of-drug addition experiment using free virus (Figure 5), L-50 exhibited a monophasic inhibition profile, with t_1/2_ of 8.2 h, similar to that exhibited by CDK-9 inhibitor DRB (t_1/2_ = 11.3 h; Figure 6B). Thus, this experiment provides strong and direct evidence that L50 does not inhibit the HIV-1 entry process and blocks Tat-dependent HIV-1 transcription, in contrast to the previously studied linear peptides derived from Tat [22], [23].

### Inhibition of reverse transcription by L50 is independent of its effects on HIV transcription

**Figure 7:**
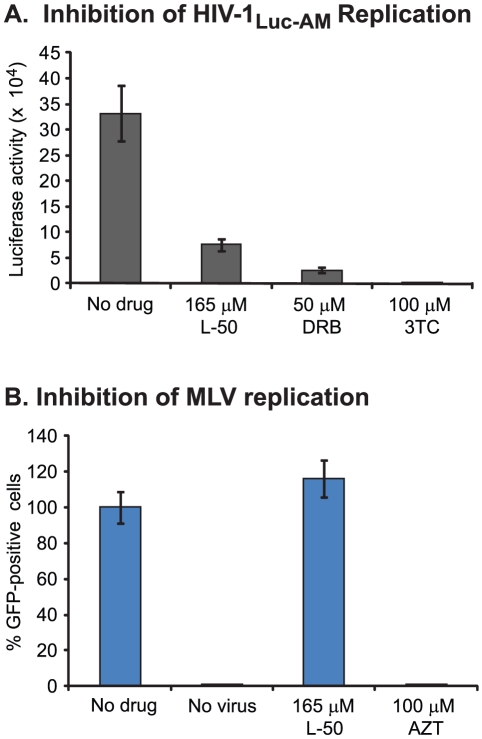
L50 effects on HIV-1 and MLV reverse transcription during cell culture infections. (**A**) HIV-1 Luc-AM has the luciferase gene under the control of the SV40 promoter within the HIV-1 genome and in place of the deleted HIV-1 env gene. This virus, pseudotyped with exogenous HIV-1 Env expression, as used to infect U87.CD4.CXCR4 cells in the absence of drugs or in the presence of L-50, DRB, or 3TC. (**B**) MLV, harboring a GFP gene and pseudotyped with VSV-G Envelope glycoprotein, was used to infect 293T cells in the absence of drugs or in the presence of L-50 or AZT.

In the experiment shown in Figure 6, we studied the late phase of L-50-mediated antiviral activity using an assay that was dependent on the Tat-TAR interaction but not on reverse transcription. To study the impact of L-50-on reverse transcription alone, we devised an assay that does not require HIV-1-dependent transcription for the readout (Figure 7A). For this experiment, we utilized an HIV-1 expression construct in which the *env* gene was replaced by a luciferase gene expressed under the control of the SV40 promoter (pNL.Luc.AM [47]). Using this reporter system, luciferase expression became dependent upon early infection events (i.e. entry, reverse transcription, integration), but the transcription of the reporter itself was independent of Tat-mediated transcriptional enhancement.

Pseudotyped HIV-1_Luc-AM_ virus was produced by co-transfecting the pNL-Luc.AM vector with pSM.WT which expresses the HIV-1_HXB2_ env glycoproteins [47]. As shown in Figure 7A, L-50 efficiently blocked luciferase expression in HIV-1_Luc-AM_ infected cells. As positive controls, both DRB and 3TC also efficiently inhibited luciferase expression from HIV-1_Luc-AM_. These findings are again consistent with a model in which L-50 blocks HIV-1 reverse transcription by binding TAR RNA.

### L-50 does not inhibit MLV reverse transcription

Reverse transcription inhibitors, such as nucleoside analogues AZT, 3TC and ddI, generally block replication of all retroviruses as well as hepatitis B virus [48]. In contrast, non-nucleoside RT inhibitors, such as nevirapine and efavirenz, specifically bind to HIV-1 RT and not to polymerases from other lentiviruses or retroviruses [48]. Thus, it is possible that L-50, binds non-specifically to RNA structures present in other retroviruses. However, as shown in Figure 7B, L-50 (250 µM) did not inhibit murine leukemia virus (MLV) whereas AZT (100 µM) completely blocked MLV replication. These data suggest that L-50 is not a general inhibitor of retroviral reverse transcription, but is specific for the HIV-1 TAR RNA sequence and/or HIV-1 reverse transcriptase.

### L-50 blocks HIV-1 reverse transcription in vitro

**Figure 8:**
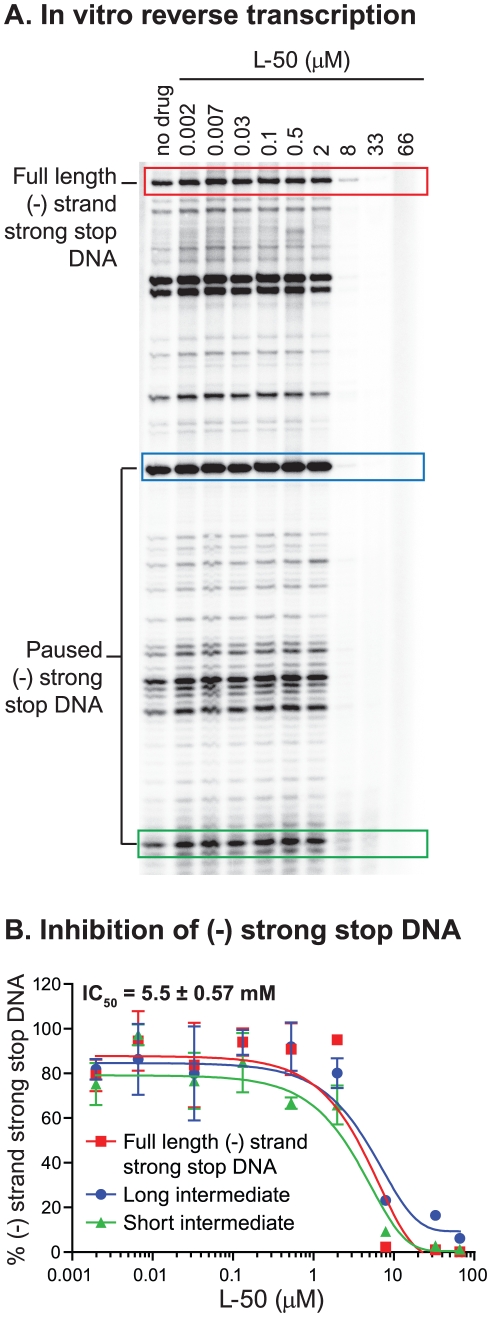
Inhibition of minus strand strong stop DNA synthesis by L50. (**A**) A reconstituted in vitro reverse transcription assay was performed with increasing L50 concentrations to determine the level of inhibition during (−) strand strong stop DNA synthesis. Quantitation using a phosphorimager was performed on full length (−) strand strong stop DNA products (full ssDNA; red box) as well as two intermediate paused DNA products (intermediate paused ssDNA; blue and green) and then plotted in panel (**B**). All experiments were performed in triplicate to calculate the L50 IC_50_ concentration (5.5±0.57 µM) to inhibit (−) strand DNA synthesis. IC_50_ values were identical for all three (−) ssDNA products.

To test directly whether L-50 is able to inhibit HIV-1 reverse transcription, we used an in vitro assay reconstituting HIV-1 reverse transcription. This assay measures the synthesis of (-) strand strong stop DNA by recombinant HIV-1 RT (p66/p51) from an 18 nt DNA primer, pre-annealed to an HIV-1 RNA template encompassing the repeat (R), 5′ unique sequence (U5), and the primer binding sequence (PBS) [49]. Addition of increasing concentrations of L-50 strongly inhibited (−) strand strong stop DNA with an IC_50_ value of 5.5±0.57 µM (Figure 8A and B). This inhibitory concentration is only slightly greater than the RNA template concentration in the reaction mixture (∼2 µM) consistent with the very tight affinity of L-50 for TAR RNA [26].

**Figure 9:**
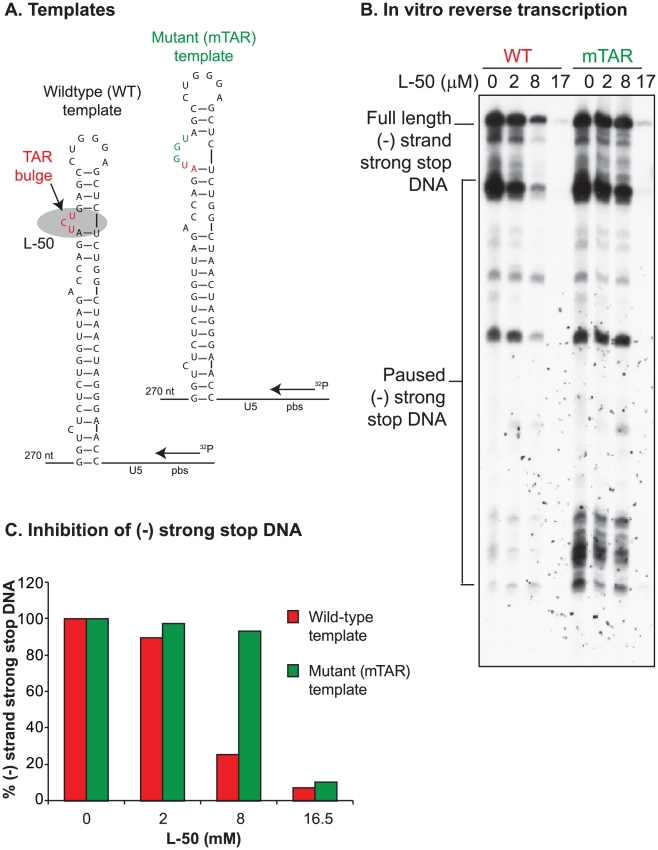
Minus strand strong stop DNA synthesis from wild type or mutant. (**A**) Schematic representation of HIV-1 RNA fragments representing the R, U5, and primer binding sequence (PBS) in wild type (wt_R_u5_pbs), site-directed mutagenesis of the TAR bulge (mutant TAR), or truncated template lacking the majority of the R region. (**B**) Minus strand DNA products catalyzed by HIV-1 RT (p66/p51) and extended from an end labelled 18 nt primer binding to the pbs. The products were resolved on an 8% denaturing PAG. (**C**) Plot of the (−) strand strong-stop DNA product produced on the various RNA templates. Bands on the gel were quantified using a phosphorimager.

To determine if L-50 inhibition of reverse transcription was dependent on the high affinity Tat-binding site located at the TAR bulge, mutations that inactivate Tat-binding were introduced into the RNA template (Figure 9A). As expected, L-50 was less potent at inhibiting the (−) strand strong stop DNA on the mutant TAR RNA template (Figure 9B and C). For example, 8 µM L-50 inhibited 80% of (−) strong stop synthesis on the wild type template but the same drug concentration inhibited less than 10% of (−) strong stop DNA synthesis on a template with mutations in the TAR L-50 binding site.

## Discussion

### Design of peptidomimetic inhibitors with high affinity for TAR RNA

The Tat-TAR complex has long been the focus of drug discovery efforts because of its central and unique role in regulating HIV-1 transcription. Unfortunately, previous attempts to discover a potent, specific and stable anti-Tat-TAR compound with efficient cellular uptake have been unsuccessful. The majority of the efforts to design Tat inhibitors involved highly charged cationic drugs [14], [15], [21], [50]–[52], oligonucleotide analogues with poor bioavailability [53], [54], or proteolytically stable peptoids [16], [17] and oligo-carbamates [55]. These compounds were abandoned due to their comparatively non-specific interactions with RNA. Other lead molecules, such as ALX40-4C [18], [22], [56] and CGP64222 [17], were initially identified as Tat-inhibitors and progressed to early clinical studies but were withdrawn from further pharmaceutical development after their antiviral activity was identified as cell surface receptor ligands.

Our design of inhibitors of the TAR RNA-Tat protein interaction started with the assumption that constrained peptidomimetics would provide effective rationale to block the relatively large interfaces formed between the viral protein and its cognate RNA [24]–[27]. The D-Pro-L-Pro template restricts pharmacophores into planar ß-hairpin structures with side chains emerging from each side. Several lead peptides, including L-20, L-50, and L-51 had nanomolar affinities for HIV-1 TAR and effectively discriminated against the analogous BIV TAR RNA sequence. Specific and high affinity binding allowed these compounds to compete with Tat protein and displace it from preformed complexes with TAR *in vitro*. This potent inhibition of Tat is remarkable considering the 3-fold reduced size of the peptidomimetics compared to the protein [25].

NMR structures of the peptide-RNA complexes revealed that Tat peptidomimetics bind in the major groove near the UCU bulge, which forms an essential part of the Tat binding site, and induce specific conformational changes that are similar to those seen with Tat [27]. Specifically, the constrained Arg residues at positions 3 and 5 of the cyclic peptide structures are located near the bulge region of TAR RNA. The pharmacophore “locking” exemplified for these two critical residues leads to remarkable specificity in binding to TAR RNA. The larger peptidomimetics show enhanced affinity due to specific interactions with the TAR RNA apical loop sequence [26].

### Cyclic peptidomimetics are potent inhibitors of HIV-1 replication

The three lead Tat peptidomimetics (L-20, L-50, and L-51) are potent inhibitors of HIV-1 replication. The lead molecule L-50 inhibits HIV-1 in primary human lymphocytes in the same concentration range as do the FDA-approved small molecule inhibitors nevirapine and 3TC. Additionally, these Tat peptidomimetics were not cytotoxic and did not inhibit cell growth up to 1 mM.

Direct addition of the peptidomimetics to cultured cells blocked HIV-1 replication, without requiring a carrier molecule to facilitate drug diffusion across plasma membranes. In contrast to previously characterized flexible peptides, which selectively inhibited HIV-1 replication by binding to the CXCR4 receptor [22], [23], L-50 inhibited a panel of both CXCR4- and CCR5-using primary HIV-1 isolates, representing the major subtypes currently circulating (A, B, C, D, and CRF01_AE). This data was satisfying because it implied that L-50 recognized conserved RNA secondary structure elements in TAR RNA even though the nucleotide sequence in this region varies considerably between strains (Figure 1D).

### Inhibition of multiple steps in the HIV-1 life cycle by the peptidomimetics

We demonstrated that the peptidomimetic L-50 inhibited Tat-mediated HIV-1 transcription using both cell free transcription assays and multiple independent *in vivo* assays using cell lines carrying reporter genes and integrated HIV-1 proviruses. In all of these assays, L-50 induced an approximately two-fold reduction in HIV-1 transcription, which was much less than the 10- to 100-fold reduction in HIV-1 replication we observed in viral replication assays. Using a variety of HIV-1 inhibitors in a time-of-drug-addition experiment we identified two specific steps in the HIV-1 lifecycle that were inhibited by L-50. As expected, L-50 was identified as an inhibitor of HIV-1 transcription since it block viral replication with a time course that closely resembled the DRB, a drug which prevents RNAP II transcriptional elongation by selectively inhibiting the protein kinase component of P-TEFb, CDK9 [43]. Additionally, L-50 inhibited an early event in HIV-1 replication. L-50 retained antiviral activity after entry inhibitor Enfuvirtide became ineffective (t_1/2_ = 1.07 h), but lost some activity (L-50 t_1/2_ = 1.8 h) approximately 4.8 hours before nucleoside RT inhibitor 3TC (t_1/2_ = 6.6 h) lost activity.

To rule out non-specific HIV-1 entry inhibition, such as had been observed for linear Tat-derived peptidomimetic inhibitors [22], [23], we used a cell-to-cell fusion assay which is not dependent upon reverse transcription or integration but faithfully mimics the mechanism of virus-to-cell binding, fusion [31] and Tat-dependent transcription (of the luciferase reporter). L-50 inhibition kinetics again coincided with DRB inhibition but there was no measurable inhibition of cell-to-cell fusion by the compound.

### Inhibition of HIV-1 reverse transcription

Both Tat and TAR RNA are thought to play an important role in HIV-1 reverse transcription [57]–[60]. TAR is a structure derived from the repeat (R) and 5′ unique sequence (U5) of the LTR, and located just upstream of the primer binding site (PBS), the initiation site of reverse transcription. There is evidence that TAR RNA structure contributes to the HIV-1 reverse transcription process [49], [61]–[64] by participating in a larger tertiary RNA secondary structure necessary for the efficient initiation of reverse transcription from tRNA^Lys,3^ [65], [66]. Nucleocapsid-mediated TAR RNA unwinding may also prevent self-priming and/or coordinate RNase H activity to free the end of single-stranded, minus strong-stop DNA. This strong-stop DNA product (complement of R and U5 RNA sequences) could then pair with the 3′ R region of RNA, adjacent to the U3 sequence, thereby facilitating the first template switch and eventual completion of minus DNA synthesis [63], [64]. This annealing process for template switching is also facilitated by the nucleocapsid protein (NC) chaperone activity [67]. Deleting or mutating TAR RNA reduces first template switch efficiency [49] and mutations that increase the stability of TAR RNA appear to block strand transfer [68].

There is also evidence that Tat plays a role in viral reverse transcription, although much of this work has been controversial. Loss of function Tat mutants have been reported to impair reverse transcription 3- to 5-fold compared to wild type HIV-1 both in infected T cells and in endogeneous reverse transcription reactions [58], [69]. Tat-mediated RNA secondary structure remodeling may facilitate obligatory strand transfers during viral DNA synthesis by reverse transcriptase [60]. However, it is possible to create replication-competent viruses where the TAR RNA sequence is replaced by a short hairpin RNA structure suggesting that Tat is dispensible for reverse transcription [70]. Our kinetic studies demonstrate that the first phase of L-50 inhibition corresponds to reverse transcriptional initiation. HIV-1 reverse transcriptase encounters the TAR stem-loop within the first ∼150 nt of minus strong-stop DNA synthesis, so the L-50-TAR RNA complex might disrupt HIV-1 reverse transcription initiation, prevent elongation or block the first template switch. Synthesis of the proviral genome (nearly 20,000 nucleotides) is thought to occur within the first 12 hours of infection, consistent with the t_1/2_ (6.6 h) we measured for 3TC (and ∼7 h reported for non-nucleoside RT inhibitor nevirapine [42]). It is then not surprising that nucleoside RT inhibitor 3TC, which blocks polymerization opposite any guanosine/deoxyguanosine base during this process, exhibits prolonged activity compared to L-50. Thus, inhibition during minus-strand strong stop DNA synthesis would likely occur immediately following entry (∼1 hrs), within the first few minutes of reverse transcription, accounting for the narrow window of L-50 efficacy in the first phase (1.8 h). AZT and 3TC blocked MLV replication whereas L-50 did not, confirming that L-50 activity is specific to HIV-1. The exact inhibitory mechanism exerted by L-50 on HIV-1 reverse transcription is the subject of ongoing studies. The data presented in Figure 8 provides in vitro evidence that L-50 blocks (−) strand strong stop DNA synthesis in a TAR-dependent manner. However, we have not ruled out additional effects of L-50 on reverse transcription, including (1) disruption of tRNA^Lys,3^ binding to HIV-1 RNA, (2) inhibition of a transition from tRNA^Lys,3^ initiation (+1 to +5 nt) to (−) strong-stop DNA elongation, (3) block in proper RNase H digestion of 5′ LTR RNA, and finally, (4) prevention of the first strand switch necessary to complete (−) strand DNA synthesis.

### Conclusions

We have shown that cyclic peptidomimetics derived from HIV-1 Tat are potent inhibitors of HIV-1 replication. Detailed characterization of the L-50 mechanism of action revealed that it is able to inhibit two important steps in the virus life cycle that involve TAR RNA: HIV-1 reverse transcription and Tat-mediated transcription. Although L-50 was more potent at inhibiting HIV-1 reverse transcription (IC_50_ = 1 to 10 µM) than HIV-1 mRNA transcription (IC_50_ >100 µM), this difference in potency might simply reflect the stoichometry necessary for inhibition. Relatively few virions enter a cell to establish infection which dramatically limits the number of TAR RNA sequences that need to be bound by L-50 (in theory, infection can be initiated by a single virion carrying only two viral genomes). By contrast, there are several thousand rounds of HIV-1 RNA transcripts produced during the later stages of the virus life cycle and each transcript carries TAR RNA as part of its leader sequence. Our unexpected discovery that L-50 has a dual inhibitory mechanism demonstrates that drugs can be designed that simultaneously inhibit reverse transcription and HIV-1 transcription by targeting the highly conserved TAR RNA element.

## Methods

### Peptide synthesis

The ADP-1 peptide containing the entire RNA-binding activity of Tat protein [71] was prepared on MBHA-Rink amide resin using Fmoc-chemistry on an Applied Biosystems 433A peptide synthesizer. The synthesis of the cyclic peptides has been described previously [25]. Peptide L-51 conjugated to a fluorescent dye was produced by coupling of commercially available 5(6)-carboxyfluorescein diacetate to an analogue of L-51, containing D-trans-4-hydrazinoproline (Hyd) in place of D-Proline (i.e. cyclo-(Arg-Thr-Arg-Thr-Arg-Gly-Lys-Arg-Arg-Ile-Arg-Val-Hyd-Pro).

### Inhibition of Tat-dependent transactivation in cell-free transcription reactions

Plasmids carrying the wild type HIV-1 LTR [71] were linearized with XbaI and biotinylated at both the 5′ and 3′ ends by incorporation of biotin-16-dUTP (Roche) [72]. In order to form the elongation complexes, the DNA was linearized, biotinylated and immobilized on streptavidin-coated magnetic beads (Dynal) that were added to the reaction mixtures as described [8]. Twenty ng of Tat protein were added and the elongation­competent complexes were assayed at increasing concentration of peptidomimetic inhibitors (usually from 0.1 to 20 µM). The reaction mixtures were incubated for 20 min at 30°C with occasional mixing and analyzed by fractionation of 6% polyacrylamide gels [8].

### Microscopy

Human fibroblasts or HeLa cells (5×104) were plated on 35 mm glass bottom culture dishes in DMEM/10% FBS (Gibco, Invitrogen) and cultured for 12 to 48 hours to obtain a confluency of 40-60%. The medium was discarded and cells were washed with PBS followed by incubation with media containing 3 mM fluorescent L-51 at 37°C for 10 min. After incubation with the fluorescent peptide, the medium was discarded and the cells were washed five times with PBS and a final volume of PBS was added for observation of living cells. Cells were imaged at 40× magnification using an Olympus IX70 epifluorescent inverted microscope. Cell observation was done with a Delta Vision RT dencovolution system and images were captured using a CCD digital camera. Scattered light was computationally reassigned using Softworx software (Applied Precision Inc.).

### Plasmids

Several previously reported eukaryotic expressioin constructs were used for this study. Plasmid pNL4.3 was originally reported by Adachi et al. [73] Plasmids pLTR.luc and pCMV.luc contains an HIV-1 LTR or CMV promoter cloned upstream of the firefly luciferase gene, respectively [74]. pDM128-LTR-fluc2 (a gift from David McDonald [75]) was produced by replacing the SV40 promoter with the HIV-1 LTR and replacing the exonic CAT coding sequence with the firefly luciferase gene. The resulting plasmid expresses firefly luciferase when the Tat dependent, LTR-driven unspliced RNA transcript is rescued by HIV-1 Rev. Plasmid pNL.luc.AM (a gift from Andre Marozsan [47]) is an HIV-1 expression construct wherein the Env ORF has been interrupted by an in-frame stop codon followed by SV40-promoted luciferase ORF. This construct produces luciferase constitutively in the transfected cells and, when co-expressed with a viral envelope supplied in trans, in target cells which have been infected by the resulting VLPs. Pseudotyped virus was produced using HIV-1 Env expression plasmid pSM.WT described elsewhere [76]. pREC-nfl (or pREC nfl_HIV-1) was cloned as previously described and lacks the 5′LTR of proviral NL4-3 DNA [45]. The near full length genomic (nfl) RNA is expressed from the CMV promoter and is spliced into all HIV-1 mRNA products to produce the full complement of HIV-1 proteins. The vector supports HIV-1 Env glycoprotein expression and cell fusion with U87.CD4.CXCR4 cells. The vector also produces virus particle that can de novo enter a susceptible cell but the core is incapable of supporting reverse transcription [45].

### Cells and viruses

U87.CD4.CXCR4/CCR5 cells were obtained through the AIDS Research and Reference Reagent Program and were maintained in DMEM (Mediatech, Inc., Herndon, PA) supplemented FBS medium (15%, Life Technologies, Inc., Rockville, MD), penicillin/streptomycin, G418 and puromycin. 293T cells were maintained in DMEM supplemented with 10% FBS and pen/strep. PBMC from HIV-seronegative donors were prepared as previously described [77]. The pNL4-3 infectious molecular clone was obtained through the AIDS Research and Reference Reagent Program. Infectious virus was produced and titered as previously described [77].

### Inhibition of Tat-dependent transactivation

293T cells were incubated with peptidomimetics and co-transfected with various combinations of pNL4-3 – an infectious molecular clone, pLTR.luc - a vector with the luciferase gene under control of the HIV-1 LTR, and pcDNA.LUC - a control plasmid expressing luciferase under control of the CMV promoter. Cells were transfected using a lipofectamine protocol as previously described [45]. Cell-free supernatant was collected 24 h post-transfection and assessed for RT activity, while the cells were lysed and assessed for luciferase activity. To assess inhibitor effects on integrated proviruses, 293T cells containing a Tat-deficient LTR reporter were pretreated with the peptides for 1 h and transfected with a Tat-expressing plasmid. The cells were incubated for 24 h, lysed, and assessed for luciferase activity.

### Viral replication/inhibition assays

Drug sensitivity assays were performed on U87.CD4.CXCR4/CCR5 cells and in peripheral blood mononuclear cells (PBMC). Peptidomimetics or control drugs were added to cells to yield final concentrations between 100 µM and 10 nM. Cells were incubated with drug for 1 h and exposed to the virus at a multiplicity of infection of 0.001, incubated with the virus for 24 h, and input virus washed away. Supernatant aliquots were removed and virus production was quantified by reverse transcriptase assay [77]. Virus production at each drug concentration was normalized and the relative values were plotted versus drug concentration to determine 50% inhibitory concentrations (IC_50_). Variations on these drug susceptibility assays are defined in each figure and related text. For the time-of-drug-inhibition experiments, HIV-1 was spinonculated onto U87-CD4/CCR5 cells as previously described [42]. The cells were washed twice with cold phosphate buffered saline (PBS) to remove unbound virions. Cells were resuspended in cold medium and split into 96-well plates (50 µl/well). Virus-cell mixes were synchronized for entry by addition of 130 µl of 37°C medium, and then AMD3100 (10 µM), Enfuvirtide/T20 (10 µM), Lamivudine/3TC (100 µM), Raltegravir (10 µM), DRB (50 µM), and L50 (250 µM) was added at one of the various time points post synchronized infection (described in Figure 5) and maintained up to 72 h. Cells were incubated for 72 h and then treated with lysis buffer and luciferase activity was determined. The same procedure was utilized for the time-of-drug-addition experiment involving cell-to-cell fusion. However, in these analyses, 293T cells were transfected with pREC.nfl [45] and then added (like virus as the method above) to the U87.CD4.CXCR4 target cells. Cell-to-cell fusion was initiated by removing cold medium and adding 37°C medium.

### In vitro reverse transcription assays

HIV-1 RNA representing the wild type R, U5, pbs, and uncoding sequence (270 nt) (wt R_U5_pbs RNA) was produced by in vitro T3 transcription (T3 MegaScript, Ambion) from a PCR product (with T3 extended primer) derived from NL4-3. The deleted R region transcript (ΔR_U5_pbs RNA) was produced using the same in vitro transcription method but from a truncated PCR production (i.e. lacking 41 nt of R region). The mutant TAR RNA transcript (with mutations 5′-UCUG-3′ to 5′-UGGU-3′ in the bulge) was generated by T3 transcription from a PCR product with the forward primer having the nt substitutions for site-directed mutagenesis. The primer sequences for PCR are available upon request. The DNA primer (18 nt), complementary to the primer binding sequence (3′ end of tRNA^Lys,3^), was annealed to the template as described [78]. The DNA primer was annealed to the various HIV-1 RNA templates by denaturation and annealing conditions previously described [78]. The primer:template annealed mixture (approximately 0.5 and 0.25 pmols) was added to 20 µl reaction mixture containing 50 ng of HIV-1 RT [49], [78] and in the absence or presence of L50 (0.002 to 66 µM). Reactions were incubated at 37°C for 45 min and then quenched with formamide EDTA loading buffer [78] for subsequent electrophoresis on a 8% denaturing polyacrylamide gel. Gels were autoradiographed and analyzed with a BioRad phosphorimager. The full length (−) strand strong stop DNA product is 181 nt on the HIV-1 wt_R_U5_pbs or mutant TAR RNA templates and 140 nt on the ΔR_U5_pbs RNA template.

## Supporting Information

Figure S1 Susceptibility of CCR5 and CXCR4-using HIV-1 isolates to L50 in either U87.CD4.CCR5 (**A**) or U87.CD4.CXCR4 (**B**) cells, respectively. The assays are described in Figure 2 of the manuscript.
(EPS)

Figure S2 Infectivity of virus treated with L50. To test the possibility that inhibition of HIV replication by L-50 is due to direct interaction of the compound with viral particles, cell-free NL4-3 HIV-1 was incubated for 2 h with or without L-50 (50 or 500 µM). After pelleting virus at 30,000g for 1 h to remove drug, NL4-3 was resuspended in 1x RPMI and used to infect U87.CD.CXCR4 cells. Cell-free supernatant was removed at day 6 to measure RT activity. The graph plots RT activity relative to the no drug control.
(EPS)
